# Дистанционное управление гликемией с применением устройств для беспроводной передачи данных у детей с сахарным диабетом 1 типа: промежуточные результаты клинической апробации

**DOI:** 10.14341/probl13492

**Published:** 2025-07-22

**Authors:** Д. Н. Лаптев, А. О. Емельянов, Е. С. Демина, И. Л. Никитина, Г. А. Галкина, А. А. Воропай, Е. С. Малышева, Ю. Г. Самойлова, В. А. Петеркова

**Affiliations:** Национальный медицинский исследовательский центр эндокринологии им. академика И.И. Дедова; Национальный медицинский исследовательский центр эндокринологии им. академика И.И. Дедова; Российская детская клиническая больница; Национальный медицинский исследовательский центр им. В.А. Алмазова; Научно-исследовательский институт акушерства и педиатрии Ростовского государственного медицинского университета Минздрава России; Научно-исследовательский институт акушерства и педиатрии Ростовского государственного медицинского университета Минздрава России; Приволжский исследовательский медицинский университет; Сибирский государственный медицинский университет Минздрава России; Национальный медицинский исследовательский центр эндокринологии им. академика И.И. Дедова

**Keywords:** сахарный диабет 1 типа у детей и подростков, СД1, телемедицина, дистанционное наблюдение, дистанционное консультирование, глюкометры с беспроводной передачей данных

## Abstract

Несмотря на заметное улучшение показателей гликемического контроля за последние годы, многие пациенты с сахарным диабетом 1 типа (СД1) не достигают целевых показателей гликированного гемоглобина (НbА1с). Учитывая необходимость регулярного взаимодействия между врачом и пациентом с СД1 и принимая во внимание географические особенности Российской Федерации, использование телемедицины может способствовать повышению эффективности проводимого лечения.

**ЦЕЛЬ:**

ЦЕЛЬ: Оценка клинической эффективности дистанционного управления гликемией с применением устройств для беспроводной передачи данных у детей с сахарным диабетом 1 типа.

**МАТЕРИАЛЫ И МЕТОДЫ:**

МАТЕРИАЛЫ И МЕТОДЫ: Критерии включения: возраст от 1 до 18 лет, диагноз «СД1», инсулинотерапия в интенсифицированном режиме (путем множественных инъекций инсулина или непрерывной подкожной инфузии инсулина). Продолжительность исследования составляла 12 месяцев, в течение которых у каждого участника проведено не менее 5 очных консультаций и не менее 8 дистанционных консультаций. Дистанционное наблюдение осуществляли с использованием мобильного приложения OneTouch Reveal (OT Reveal).

**РЕЗУЛЬТАТЫ:**

РЕЗУЛЬТАТЫ: Всего в исследование было включено 58 пациентов с СД1 в возрасте от 1 до 18 лет. Уровень HbA1c за период исследования снизился с 7,6% [7,0; 8,7] исходно до 7,2% [6,5; 8,2] к концу исследования, через 12 месяцев наблюдения (p=0,025). Следует отметить, что наилучший гликемический контроль отмечался через 3 месяца наблюдения (HbA1c — 7,2% [6,5; 8,5], число детей с HbA1c<7,0% — 44% (31-59)). Медиана % измерений в целевом диапазоне от 3,9 до 10,0 ммоль/л увеличилась с 51,1% [38,9; 63,6] исходно до 59,6% [46,9; 69,8] (p=0,03).

**ЗАКЛЮЧЕНИЕ:**

ЗАКЛЮЧЕНИЕ: Использование глюкометра в комбинации с программным обеспечением для управления сахарным диабетом и возможностью дистанционной передачи данных сопровождается существенным улучшением гликемического контроля у детей с СД1. Дальнейшие результаты клинической апробации позволят детальнее оценить эффективность такого подхода, однако уже сейчас можно сказать, что более широкое использование этих возможностей позволит повысить доступность медицинской помощи и добиться лучшей компенсации у многих пациентов с СД1.

## Abstract (en)

Despite marked improvements in glycemic control in recent years, many patients with type 1 diabetes mellitus (T1DM) do not achieve glycated hemoglobin (HbA1c) targets.Considering the need for regular interaction between a doctor and a patient with T1DM, and taking into account the geographical features of the Russian Federation, the use of telemedicine can help improve the effectiveness of treatment.

**OBJECTIVE:**

OBJECTIVE: To evaluate the clinical effectiveness of remote glycemic control using devices for wireless data transmission in children with type 1 diabetes mellitus.

**MATERIALS AND METHODS:**

MATERIALS AND METHODS: Inclusion criteria: Age from 1 to 18 years, diagnosis of T1DM, intensive insulin therapy (through multiple insulin injections or continuous subcutaneous insulin infusion). The duration of the study was 12 months, during which each participant had at least 5 face-to-face consultations and at least 8 remote consultations. Remote monitoring was performed using the OneTouch Reveal (OT Reveal) mobile application.

**RESULTS:**

RESULTS: A total of 58 patients with T1DM aged 1 to 18 years were included in the study. The HbA1c level during the study period decreased from 7.6% [7.0; 8.7] at baseline to 7.2% [6.5; 8.2] by the end of the study, after 12 months of follow-up (p=0.025). It should be noted that the best glycemic control was observed after 3 months of observation (HbA1c —7.2% [6.5; 8.5], number of children with HbA1c<7.0% — 44% (31–59)). The median % of measurements in the target range from 3.9 to 10.0 mmol/L increased from 51.1% [38.9; 63.6] at baseline to 59.6% [46.9; 69.8] (p=0.03).

**CONCLUSION:**

CONCLUSION: Using a glucose meter in combination with diabetes management software and remote data transfer capabilities is associated with significant improvements in glycemic control in children with T1DM. Further results of clinical testing will allow a more detailed assessment of the effectiveness of this approach, but it can already be said that wider use of these opportunities may improve the availability of medical care and achieve better compensation for many patients with T1DM.

Несмотря на заметное улучшение показателей гликемического контроля за последние годы, многие пациенты с сахарным диабетом 1 типа (СД1) не достигают целевых показателей гликированного гемоглобина (НbА1с). Так, по данным Федерального регистра сахарного диабета, лишь 33% детей и 20% подростков с СД1 имеют показатели НbА1с менее 7%, при этом у более чем 20% пациентов регистрируется НbА1с более 9% [1]. Превышение целевых показателей гликемического контроля значительно повышает риск развития и прогрессирования хронических осложнений СД1.

Учитывая необходимость регулярного взаимодействия между врачом и пациентом с СД1 и принимая во внимание географические особенности Российской Федерации (РФ), использование телемедицины может способствовать повышению эффективности проводимого лечения. В последние годы информационные технологии в секторе здравоохранения стали более доступными. И несмотря на то, что телемедицина в РФ имеет ограниченное распространение, из этого способа предоставления медицинских услуг можно извлечь много дополнительной потенциальной выгоды, как для пациента, так и Для системы здравоохранения в целом. Телемедицина может помочь устранить ряд проблем в системе здравоохранения, таких как своевременное предоставление медицинских ресурсов (например, специалистов), минимизировать ограничения, связанные с перемещением пациентов, обеспечить экономию времени и средств.

На сегодняшний день получены и представлены отдельные результаты клинического использования телемедицины у детей с СД1 [2–7]. Вместе с тем остаются не ясными результаты системного применения данного подхода в рамках оказания медицинской помощи.

В работе представлены промежуточные результаты клинической апробации метода дистанционного управления гликемией с применением устройств для беспроводной передачи данных у детей с СД1.

## ЦЕЛЬ

Оценка клинической эффективности дистанционного управления гликемией с применением устройств для беспроводной передачи данных у детей с СД1.

## МАТЕРИАЛЫ И МЕТОДЫ

## Место и время проведения исследования

Исследование выполнено с января 2022 по декабрь 2023 гг. на базе следующих клинических центров: ФГБУ «НМИЦ эндокринологии» Минздрава России, РДКБ ФГАОУ ВО РНИМУ им. Н.И. Пирогова МЗ РФ, ФГБОУ ВО СибГМУ Минздрава России, ФГБОУ ВО НИИАП РостГМУ Минздрава России, ФГБУ НМИЦ им. В.А. Алмазова, ФГБОУ ВО «ПИМУ» Минздрава России.

## Дизайн исследования

Проспективное, многоцентровое, сравнительное исследование.

## Изучаемые популяции и способ формирования выборки

Отбор пациентов осуществлялся из лиц, обратившихся в вышеуказанные центры, на основании установленных критериев включения, невключения и исключения.

Критерии включения: возраст от 1 до 18 лет, диагноз «СД1», инсулинотерапия в интенсифицированном режиме (путем множественных инъекций инсулина или непрерывной подкожной инфузии инсулина), полученное письменно информированное согласие родителей на участие в исследовании.

Критерии невключения: отсутствие возможности самостоятельно (пациентом или родителем ребенка с СД1) проводить самоконтроль гликемии с помощью выдаваемого в рамках исследования индивидуального глюкометра с необходимой частотой; отсутствие технической возможности дистанционной передачи результатов измерений (проживание в зоне устойчивого покрытия сети Интернет, отсутствие совместимого с программным обеспечением смартфона и др.); наличие у пациента форм и/или особенностей течения заболевания, при которых достоверную информацию о состоянии его здоровья невозможно получить при дистанционном наблюдении.

Критерии исключения: показатель HbA1c на момент скрининга <7% или >12%; невозможность следовать требованиям протокола клинической апробации, отзыв информированного добровольного согласия; неустранимые технические проблемы, связанные с оборудованием для проведения дистанционного мониторинга или с его использованием пациентом; отсутствие фактического проведения измерений пациентом в течение 7 дней или более 20% дней в календарном месяце; отсутствие возможности дистанционного контакта с пациентом; невыполнение пациентом рекомендаций, полученных в ходе дистанционного наблюдения.

## Целевые показатели исследования

Основной показатель исследования

Изменение HbA1c к концу исследования по сравнению с исходным уровнем и доля пациентов, достигших HbA1c менее 7,0% к концу исследования.

Дополнительные показатели исследования

Изменение к концу исследования по сравнению с исходным уровнем: 1) показателей гликемического контроля: процент измерений в диапазоне от 3,9 до 10,0, менее 3,9 и более 10,0 ммоль/л.

Описание медицинского вмешательства

Продолжительность исследования составляла 12 месяцев, в течение которых у каждого участника проведено не менее 5 очных консультаций и не менее 8 дистанционных консультаций. Дистанционное наблюдение осуществляли с использованием мобильного приложения OneTouch Reveal (OT Reveal).

Пациенту или его законному представителю было рекомендовано выполнять измерения глюкозы крови с частотой не менее 4 раз в сутки (перед едой, через 2 часа после еды, перед сном, периодически ночью) с помощью совместимого с программным обеспечением индивидуального глюкометра OneTouch Verio Reflect. Лечащий врач пациента через свою учетную запись в программном обеспечении не реже одного раза в месяц просматривал и анализировал отчеты. На основании полученных данных проводилось дистанционное консультирование и при необходимости корректировалась проводимая терапия. Дистанционное консультирование в зависимости от полученных данных отчета могло проводиться в режиме отложенных консультаций (без видеоконференцсвязи) или в режиме «реального времени» с видеоконференцсвязью.

Частота просмотра/анализа данных отчетов и дистанционного консультирования могла определяться лечащим врачом индивидуально, но не реже одного раза в месяц.

## Методы

Всем пациентам на очном визите проводили стандартный осмотр и антропометрию, осуществляли исследование уровня HbA1c, регистрацию и анализ показателей гликемии и суточных доз инсулина, оценку и коррекцию проводимого лечения, давали рекомендации по дальнейшему самоконтролю и наблюдению.

OneTouch Reveal (OT Reveal)

Методика дистанционного наблюдения с мобильным приложением подробно описана нами ранее [4]. Вкратце: мобильное приложение OT Reveal представляет собой дневник самоконтроля в электронном формате с автоматическим построением структурированных отчетов и возможностью направлять их в электронном виде лечащему врачу. Также лечащий врач имел доступ к данным пациента через профессиональное приложение с веб-интерфейсом. На основании отчетов врачи проводили дистанционное консультирование с коррекцией проводимой терапии, обучение по самоконтролю.

## Методы измерения целевых показателей

HbA1c

Исследование уровня HbA1c проводили методом реакции агглютинации моноклональных антител на анализаторе DCA Vantage Analyzer (Siemens, Германия) или методом жидкостной ионообменной хроматографии на анализаторе BioRad D10 (BioRad, США). Регистрацию показателей гликемии осуществляли по данным, полученным с помощью мобильного приложения OT Reveal.

## Статистические процедуры

Принципы расчета размера выборки: размер выборки предварительно не рассчитывали.

Пропущенные значения

Пропущенные значения заполняли на основании медианы по каждой выделенной группе отдельно.

Статистические методы

Статистическая обработка проводилась в Python 3.10.2 с использованием открытой библиотеки SciPy 1.11.3 [8].

Совокупности количественных показателей описывали при помощи значений медианы (Me), нижнего и верхнего квартилей (Q1–Q3). Различие между количественными признаками в зависимых выборках оценивали с помощью критерия Фридмана. Номинативные данные описывались в виде абсолютной (n) и относительной частоты (%). Доверительный интервал (ДИ) для биноминальных пропорций (частот) рассчитывался методом Уилсона с поправкой на непрерывность [9]. Для сравнения частот в изучаемых группах использовались таблица кросстабуляции и статистика χ² с поправкой на непрерывность, когда это необходимо (количество ожидаемых наблюдений в любой из ячеек <5). Статистически значимыми считались различия при p<0,05.

Вариабельность гликемии оценивали по коэффициенту вариации — CV (отношение стандартного отклонения к среднему).

Статистически значимыми считали различия при p<0,05.

## Этическая экспертиза

Протокол исследования одобрен локальным комитетом по этике. До включения в исследование законные представители пациентов, а с 15-летнего возраста и пациенты подписали информированное согласие на участие в нем.

## РЕЗУЛЬТАТЫ

Всего в исследование было включено 58 пациентов с СД1 в возрасте от 1 до 18 лет. Во всей выборке медиана возраста составила 11,8 года [ 10,0; 13,2], длительности заболевания — 4,9 года [ 2,3; 7,6], уровня гликированного гемоглобина — 7,6 % [ 7,0; 8,7].

## Показатели HbA1c за весь период исследования

Уровень HbA1c за период исследования снизился с 7,6% [ 7,0; 8,7] исходно до 7,2% [ 6,5; 8,2] к концу исследования, через 12 месяцев наблюдения (p=0,025) (рис. 1). Снижение уровня НbА1с сопровождалось увеличением числа пациентов, достигших компенсации заболевания: доля пациентов с HbA1c<7,0% увеличилась с 19% (10-32) до 35% (17-57) (χ²=9,5; p=0,049), в то же время доля пациентов с HbA1c>9,0% снизилась с 29% (16-39) до 12% (3-31) (χ²=11; p=0,027). Следует отметить, что наилучший гликемический контроль отмечался через 3 месяца наблюдения: число детей с HbA1c<7,0% — 44% (31–59)).

**Figure:**
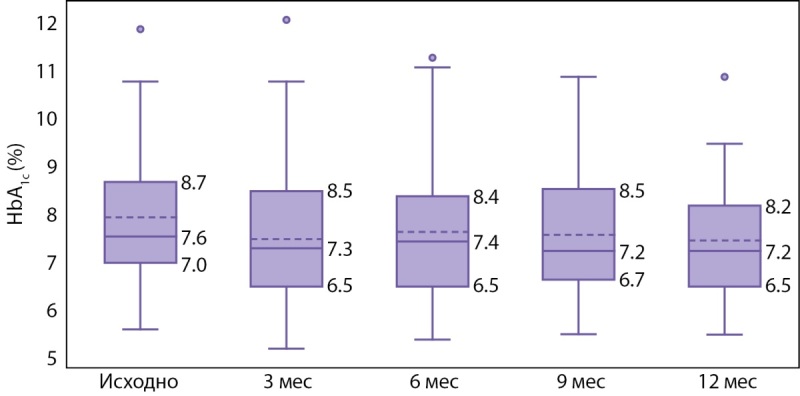
Рисунок 1. Показатели гликированного гемоглобина (НbА1с) в течение исследования. Данные на графике: медиана (—), среднее (---), интерквантильный диапазон (£). Для сравнения НbА1с использован критерий Фридмана: статистическая величина = 11,2, p=0,025.

## Распределение показателей гликемии по диапазонам

Параллельно со снижением уровня гликированного гемоглобина отмечалось увеличение % измерений в целевом диапазоне от 3,9 до 10,0 ммоль/л, что в основном достигалось за счет снижения % измерений выше целевого диапазона более 10,0 ммоль/л (рис. 2).

**Figure:**
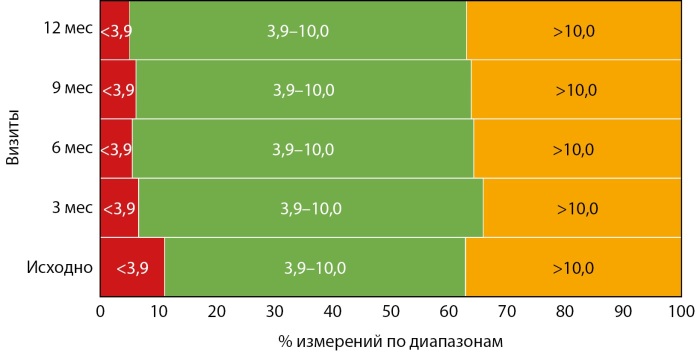
Рисунок 2. Распределение показателей глюкозы по диапазонам в зависимости от визита (средние значения).

Медиана % измерений в целевом диапазоне от 3,9 до 10,0 ммоль/л увеличилась с 51,1% [ 38,9; 63,6] исходно до 59,6% [ 46,9; 69,8] (p=0,03). При этом медиана % измерений выше целевого диапазона более 10,0 ммоль/л значимо не изменилась: исходно — 35,5% [ 23,5; 45,8], через 12 месяцев — 30,9% [ 21,9; 51,1] (p=0,2), как и медиана % измерений менее 3,9 ммоль/л: исходно — 5,0 [ 2,1; 20,1], через 12 месяцев — 5,1 [ 2,0; 8,0] (p=0,5).

## Другие показатели

Показатели вариабельности гликемии, определяемые по показателю коэффициента вариации (CV), статистически значимо не изменились за время исследования и составили: 39,2% [ 32,5; 46,1] исходно и 36,5% [ 31,2; 44,6] по окончании исследования (p=0,8).

## ОБСУЖДЕНИЕ

Дистанционные методы взаимодействия врача и пациента (телемедицинские технологии) в последние годы стали значительно шире использовать во всех странах, включая Россию. Результаты многих исследований показывают, что использование средств телемедицины в лечении сахарного диабета позволяет эффективнее достигать терапевтических целей лечения большему числу пациентов, а также существенно оптимизирует работу врача (более обоснованные изменения терапевтической схемы лечения, более частая коррекция схемы лечения, меньше времязатрат и трудозатрат на выработку правильной терапевтической тактики в отношении каждого пациента) [2–4].

«Облачное» программное обеспечение собирает данные из глюкометров и предоставляет проанализированные показатели, помогающие как пациентам, так и лечащему врачу принимать более информированные решения в отношении лечения и образа жизни.

Так, в недавно опубликованном исследовании [10] проанализировали обезличенные показатели гликемии, выгруженные с сервера мобильного приложения для контроля диабета, более чем 55 000 больных с СД. Пациенты проводили самоконтроль с помощью глюкометра, совместимого с данным приложением, с частотой в среднем не реже 1 раза в сутки на протяжении 180 дней. Данные каждого субъекта, полученные в первые 14 дней использования приложения с глюкометром, попарно сравнили с данными того же субъекта, полученными за последние 14 дней перед окончанием 180-дневного периода использования приложения. У пациентов с СД1 и СД2 через 180 дней % измерений в целевом диапазоне 3,9–10,0 ммоль/л увеличился с 57,9% до 65,7% и с 72,8% до 84,8%, средний уровень глюкозы уменьшился на -0,8 ммоль/л и -1,1 ммоль/л соответственно по сравнению с исходным уровнем без клинически значимых изменений количества эпизодов гипогликемии. Среди пациентов с СД1, проводивших от двух до четырех сеансов или от 10 до 20 минут в неделю в приложении, % измерений в целевом диапазоне 3,9–10,0 ммоль/л улучшился на 7,0 и 8,2 процентного пункта соответственно. Среди пациентов с СД2, проводивших от двух до четырех сеансов или от 10 до 20 минут в неделю в приложении, % измерений в целевом диапазоне 3,9-10,0 ммоль/л улучшился на 12,6 и 12,1 процентного пункта соответственно. Все изменения гликемии были статистически значимыми (р<0,0005). Безусловно, данные, полученные в условиях реальной клинической практики, часто имеют ограничения, связанные с осведомленностью об анамнезе пациентов, приверженности лечению, коррекции терапии сахарного диабета в течение периода исследования, индивидуальных целях гликемического контроля и другие. Тем не менее исследователи установили клинически значимое улучшение % измерений в целевом диапазоне 3,9–10,0 ммоль/л с пропорциональным и клинически значимым снижением % измерений в диапазоне гипергликемии среди тех пациентов, кто начал использовать мобильное приложение для контроля диабета и совместимый с ним глюкометр.

«Облачное» программное обеспечение для контроля диабета (cloud-connected diabetes application) позволяет лечащему врачу наблюдать за течением заболевания у пациентов и создает возможность дистанционного консультирования.

В нашем исследовании показано, что наблюдение детей с СД1 с использованием мобильного приложения для дистанционного контроля за гликемией позволяет улучшить и поддерживать на этом уровне в течение длительного времени показатели гликемического контроля, включая уровень HbA1c и % измерений в целевом диапазоне 3,9–10,0 ммоль/л.

Схожие результаты были получены и в других исследованиях с использованием «облачного» программного обеспечения для контроля диабета и дистанционной передачи данных.

Например, в сравнительном исследовании [11] было рандомизировано 120 пациентов с СД1 и СД2 с исходным средним уровнем HbA1c 9,6% для использования обычного глюкометра без беспроводной передачи данных (контрольная группа) или глюкометра с беспроводной передачей данных в сочетании с приложением (группа наблюдения) в течение 12 недель. Через 12 недель пациентам из контрольной группы было предложено использовать глюкометр с беспроводной передачей данных в сочетании с приложением еще в течение 12 недель; пациенты из группы наблюдения продолжили использовать глюкометр и приложение, чтобы оценить устойчивость результата. Пациентам из группы «глюкометр + приложение» было запланировано получение текстовых сообщений, касающихся сахарного диабета, от лечащих врачей каждые 2 недели. Клинические показатели и регистрируемые самими пациентами параметры оценивались в ходе непосредственных визитов в клинику во время общения между участником и лечащим врачом исходно, через 12 и 24 недели. Снижение уровня HbA1c через 12 недель по сравнению с исходным уровнем имело место как в контрольной группе (обычный глюкометр) (n=36) (-0,63%, P<0,01), так и в группе наблюдения («глюкометр + приложение») (n=74) (-0,99%, P<0,001). Разность в снижении HbA1c между двумя группами через 12 недель была статистически значимой (-0,36%, P=0,045). После того, как на 12-й неделе пациенты из контрольной группы были переведены на «глюкометр + приложение», в течение следующих 12 недель наблюдалось дальнейшее весьма значимое снижение уровня HbA1c на −0,55% (P<0,001). В то же время у пациентов из группы наблюдения произошло дополнительное снижение HbA1c на −0,16%, однако эта разница не была статистически значимой (P>0,05).

В ряде исследований у детей с СД1 также показана успешность использования «облачного» программного обеспечения для дистанционной оценки гликемических профилей и телемедицинского наблюдения [2–4]. По данным этих работ, использование «облачного» программного обеспечения для дистанционного наблюдения позволяет добиться снижения уровня HbA1c (в зависимости от критериев включения, от 0,5% до 5,0%), увеличения числа пациентов, достигнувших компенсации заболевания (в зависимости от критериев включения, от 16 до 60%), увеличения % измерений в целевом диапазоне (в зависимости от критериев включения, от 57,2 до 83,6%), а также улучшения качества жизни и удобства самоконтроля у детей с СД1. В то же время многие из этих работ были направлены на целевые популяции (например, впервые выявленный СД1, подростки и др.), что осложняло распространение результатов на всех детей с СД1. Проведение клинической апробации у детей с СД1 в возрасте от 1 до 18 лет с использованием «облачного» программного обеспечения позволит получить необходимые данные для подтверждения раннее полученных результатов для дальнейшего внедрения в практическое здравоохранение.

## ЗАКЛЮЧЕНИЕ

Использование глюкометра в комбинации с программным обеспечением для управления сахарным диабетом и возможностью дистанционной передачи данных сопровождается существенным улучшением гликемического контроля у детей с СД1. Дальнейшие результаты клинической апробации позволят детальнее оценить эффективность такого подхода, однако уже сейчас можно сказать, что более широкое использование данных возможностей может позволить повысить доступность медицинской помощи и добиться лучшей компенсации у многих пациентов с СД1.

## ДОПОЛНИТЕЛЬНАЯ ИНФОРМАЦИЯ

Финансирование проекта. Работа выполнена в рамках клинической апробации метода дистанционного управления гликемией с применением устройств для беспроводной передачи данных у детей с сахарным диабетом 1 типа по сравнению с традиционным диспансерным наблюдением.

Конфликт интересов. Авторы декларируют отсутствие явных и потенциальных конфликтов интересов, связанных с публикацией настоящей статьи.

Участие авторов. Петеркова В.А. — научное руководство, дизайн и планирование исследования; Лаптев Д.Н. — выгрузка данных из регистра, анализ и статистическая обработка полученных данных, написание текста; Емельянов А.О. — ведение регистра пациентов, выгрузка данных из регистра, написание и редактирование текста; Демина Е.С. — ведение регистра пациентов, выгрузка данных из регистра; Никитина И.Л. — ведение регистра пациентов, выгрузка данных из регистра; Галкина Г.А. — ведение регистра пациентов, выгрузка данных из регистра; Воропай А.А. — ведение регистра пациентов, выгрузка данных из регистра; Малышева Е.С. — ведение регистра пациентов, выгрузка данных из регистра; Самойлова Ю.Г. — научное руководство, дизайн и планирование исследования.
